# New Opioid Use and Risk of Emergency Department Visits Related to Motor Vehicle Collisions in Ontario, Canada

**DOI:** 10.1001/jamanetworkopen.2021.34248

**Published:** 2021-11-11

**Authors:** Qi Guan, Daniel McCormack, David N. Juurlink, Susan E. Bronskill, Hannah Wunsch, Tara Gomes

**Affiliations:** 1 Institute of Health Policy, Management and Evaluation, University of Toronto, Toronto, Ontario, Canada; 2 ICES, Toronto, Ontario, Canada; 3 Department of Medicine, University of Toronto, Toronto, Ontario, Canada; 4 Sunnybrook Research Institute, Toronto, Ontario, Canada; 5 Department of Critical Care Medicine, Sunnybrook Health Sciences Centre, Toronto, Ontario, Canada; 6 Department of Anesthesiology and Pain Medicine, University of Toronto, Toronto, Ontario, Canada; 7 Department of Anesthesiology, Columbia University Medical College, New York, New York; 8 Interdepartmental Division of Critical Care Medicine, University of Toronto, Toronto, Ontario, Canada; 9 Li Ka Shing Knowledge Institute, St Michael’s Hospital, Ontario, Toronto, Canada; 10 Leslie Dan Faculty of Pharmacy, University of Toronto, Toronto, Ontario, Canada

## Abstract

**Importance:**

Opioids can impair motor skills and may affect the ability to drive; however, the association of opioid use with driving ability is not well established.

**Objective:**

To examine the risk of motor vehicle collisions (MVCs) among drivers starting opioid therapy compared with that among drivers starting nonsteroidal anti-inflammatory drug (NSAID) therapy.

**Design, Setting, and Participants:**

This population-based, retrospective cohort study included all residents of Ontario aged 17 years or older who started new prescription analgesic therapy between March 1, 2008, and March 17, 2019.

**Exposures:**

Initiation of opioid therapy or NSAID therapy, ascertained through prescription dispensing records in administrative data.

**Main Outcomes and Measures:**

The primary outcome was an emergency department visit for injuries sustained as a driver in an MVC during the 14 days after starting analgesic therapy. Inverse probability treatment weighting was used to balance baseline covariates, and weighted Cox proportional hazards regression models were used to assess the association between new analgesic therapy and hazard of an emergency department visit after an MVC.

**Results:**

Of the 1 454 824 individuals included in the study, 765 464 (52.6%) were new opioid recipients and 689 360 (47.4%) were new NSAID recipients. Most participants were aged 65 years or older (75.2%), and 55.2% were women. Of 194 individuals who had emergency department visits for injuries from an MVC within 14 days of initiating therapy, 98 (50.5%) were opioid recipients (3.41 per 1000 person-years; 95% CI, 2.80-4.15 per 1000 person-years) and 96 (49.5%) were NSAID recipients (3.64 per 1000 person-years; 95% CI, 2.98-4.45 per 1000 person-years). There was no significant difference in the risk of an emergency department visit for MVC injuries between opioid and NSAID recipients (weighted hazard ratio, 0.94; 95% CI, 0.70-1.25).

**Conclusions and Relevance:**

The findings of this study suggest that the hazard of an emergency department visit for injuries relating to an MVC as a driver is similar between individuals starting prescription opioids and those starting prescription NSAIDs. These results may be useful for patients, clinicians, and caregivers when considering new analgesic therapy.

## Key Points

**Question:**

Is initiation of prescription opioid therapy associated with a higher hazard of an emergency department (ED) visit after a motor vehicle collision compared with initiation of prescription nonsteroidal anti-inflammatory drug (NSAID) therapy?

**Findings:**

In this cohort study of 1 454 824 individuals with new analgesic exposure, there was no significant difference in the hazard of an ED visit after a motor vehicle collision between opioid and NSAID recipients; however, the rate of collisions was higher among drivers receiving new analgesic therapy than in the general population.

**Meaning:**

The findings suggest that after initiation of analgesic therapy, the hazard of an ED visit for injuries related to a motor vehicle collision is similar for opioid and NSAID recipients.

## Teaser

This cohort study compares the risk of an emergency department (ED) visit after a motor vehicle collision among drivers initiating prescription opioid therapy with that among drivers initiating prescription nonsteroidal anti-inflammatory drug therapy.

## Introduction

Motor vehicle collisions (MVCs) are among the leading causes of unintentional morbidity and mortality in North America.^1,2^ In 2018, there were 414 visits to an emergency department (ED) for injuries resulting from MVCs per 100 000 population in Canada^3^ and 1058 per 100 000 population in the United States.^4^ The cause of MVCs is multifactorial, but a potential contributor is use of psychoactive medications such as prescription opioids. Opioids can cause somnolence and slow reaction times, and clinicians have been concerned about their effects on driving ability for decades.^5,6,7,8^ The prevalence of prescription opioid use in Canada is high, with 1 in 8 individuals receiving a prescription in 2018^9^; therefore, it is important to fully understand the potential association between prescription opioid use and risk of MVCs.

Epidemiologic studies have shown associations between opioid use and MVC risk in some scenarios, such as a dose-dependent increase in risk^8^ and increased risk when opioids are used with other psychoactive substances^10^; however, the extent of the potential contribution of new opioid use to MVC risk remains uncertain. In this study, we examined the association of new opioid use with hazard of an ED visit related to an MVC with new use of nonsteroidal anti-inflammatory drugs (NSAIDs) as an active comparator because of the minimal psychoactive effects of NSAIDs.^11,12^ Previous studies have shown that collisions associated with NSAID exposure are uncommon and are more likely associated with the underlying condition that prompted NSAID therapy.^11,12^ Thus, NSAIDs make an ideal real-world comparison drug for opioids when evaluating the potential effects of the psychoactive properties of opioids. The objective of this study was to compare the short-term risks of ED visits for injuries from an MVC between drivers initiating prescription opioid therapy and drivers initiating NSAID therapy to differentiate the potential risk between the 2 prescription analgesic options.

## Methods

### Setting

We conducted a population-based, retrospective cohort study among all individuals of driving age in Ontario, Canada, who started treatment with a prescription opioid or prescription NSAID between March 1, 2008, and March 17, 2019. Ontario is the most populous and ethnically diverse province in Canada, and all residents (>14 million as of 2020^13^) have access to universal coverage for physician and hospital services. Data used in this study were held in databases at ICES^14^ (formerly known as the Institute for Clinical Evaluative Sciences) in Toronto, Ontario, Canada. These data sets were linked using unique encoded identifiers and analyzed at ICES. Use of these data was authorized under §45 of the Ontario Personal Health Information Protection Act, which does not require review by a research ethics board or informed consent. This study followed the Strengthening the Reporting of Observational Studies in Epidemiology (STROBE) reporting guideline.

### Data Sources

We used the Ontario Drug Benefits database to identify records of prescription medications dispensed to individuals in this study. Provincially funded medications are available through the Ontario Drug Benefits program to residents who have low income status, are aged 65 years or older, live in a long-term care facility, receive home care, or experience high drug costs relative to their income. Between January 1, 2018, and March 31, 2019, Ontario Drug Benefits also provided drug coverage for all residents of Ontario younger than 25 years. We used the Canadian Institutes of Health Information National Ambulatory Care Reporting System to identify diagnoses and procedures that occurred during ED visits and the Canadian Institutes of Health Information Discharge Abstract Database to identify corresponding measures during inpatient hospitalizations. We used the Ontario Health Insurance Plan Database to obtain records regarding outpatient physician services and the Registered Persons Database to obtain patient demographic information.

### Study Cohort

The cohort consisted of individuals aged 17 years or older who filled a prescription for an opioid or NSAID paid for by the province between March 1, 2008, and March 17, 2019. The dispensing date was deemed the index date. We only included the first prescription opioid or NSAID claim during the study period to maintain independent events. To limit the cohort to patients initiating analgesic therapy, we excluded patients who were dispensed an opioid or NSAID in the preceding 12 months. We excluded those who received palliative care (eTable 1 in the Supplement gives codes) during the 6 months before the index date and those who lived in a long-term care facility on or before the index date because these individuals were less likely to drive a motor vehicle. In addition, opioids are often prescribed in such settings in a manner different from that used with patients requiring analgesics for acute pain in outpatient settings. We excluded individuals whose index date overlapped a hospitalization and those who were recently (within 2 days) discharged from the hospital to avoid potential inpatient analgesic exposure. We excluded individuals who received both a prescription opioid and a prescription NSAID on the index date and those with an ED visit related to injuries from an MVC in the week before the index date to ensure that any outcomes measured were associated with new MVCs and not complications from injuries associated with earlier MVCs that may have prompted analgesic therapy (Figure).

**Figure.**
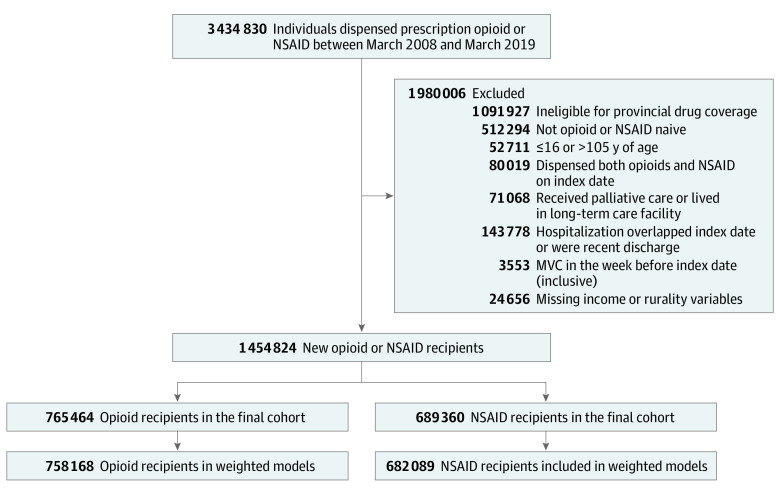
Flowchart of Individuals Included in the Study MVC indicates motor vehicle collision; NSAID, nonsteroidal anti-inflammatory drug.

### Outcomes

Our outcome of interest was any ED visit for injuries sustained as a driver in an MVC in the 14 days after the index date (date on which first prescription opioid or NSAID was dispensed). We also replicated the study using the outcome of ED visits for injuries sustained as a passenger in an MVC during the same period (eTable 2 in the Supplement shows driver and passenger external cause-of-injury codes) as a sensitivity analysis, with the expectation that no significant differences would be observed in the rate of MVC injuries as a passenger between opioid recipients and NSAID recipients.

### Statistical Analysis

We summarized baseline characteristics of the cohort using descriptive statistics and compared the 2 exposure groups (opioid recipients vs NSAID recipients) using standardized differences. A comparison was considered as meaningfully different when the standardized difference was greater than 0.10.^15^

To account for potential differences in the distribution of baseline covariates between the opioid and NSAID groups, we used inverse probability treatment weighting (IPTW). These weights were estimated using propensity scores and represent the reciprocal of the probability of receiving the treatment that the individual actually received. The propensity score was estimated by regressing the exposure on a number of covariates using a logistic regression, including age; sex; rurality; income quintile; Adjusted Clinical Groups score from the Johns Hopkins Adjusted Clinical Groups System (version 10.0)^16^; diagnosis of alcohol use disorder or other substance use disorders in the past year (eTable 1 in the Supplement shows diagnosis codes); use of medications with warnings regarding driving and operating heavy machinery in Canada (ie, angiotensin II receptor blockers, antihistamines, barbiturates, benzodiazepines, monoamine oxidase inhibitors, or phenothiazines); number of outpatient, inpatient, or ambulatory physician visits in the past year; and number of ED visits within the past year for injuries related to an MVC.

When we applied the stabilized weights to the cohort and checked for balance between the exposure groups using weighted standardized differences, some characteristics remained unbalanced. Therefore, we trimmed the cohort by removing individuals within the highest and lowest 0.5 percentile of IPTW to achieve balance (eFigure in the Supplement). Weighted cohort characteristics are presented in eTables 4 and 5 in the Supplement.

We used weighted Cox proportional hazards regression models to evaluate the association between new prescription analgesic therapy and the hazard of an ED visit for injuries related to an MVC among drivers. We adjusted for fiscal year in all models to account for potential changes in prescribing habits that occurred during the past decade. In a secondary analysis, we stratified opioid recipients into groups of individuals whose initial daily prescription opioid dose was less than 50 mg of morphine or equivalent (MEQ) and those with an initial opioid dose of 50 MEQ or greater per day to explore potential dose response. For this calculation, we used the formula described by the Ontario Drug Policy Research Network: dose = quantity × strength × conversion factor.^17^ This formula converts the dose of all nonmorphine opioids to morphine equivalents using the strength and quantity listed on the prescription record as well as its morphine equivalency conversion factor. The final dose value was then divided by the number of days supplied (indicated on the prescription claim) to identify the daily dose. If multiple opioid prescriptions were dispensed at initiation of therapy, the daily dose of each prescription was calculated and then added for a total initial daily dose. The 50-MEQ dose threshold was chosen based on the most recent Canadian Guideline for Opioid Therapy and Chronic Noncancer Pain.^18^ We report weighted hazard ratios and 95% CIs.

We tested for violation of the proportional hazards assumption by examining the plot of the log of the negative log of survival function estimates vs the log of survival time and by adding a time-dependent covariate representing the interaction of exposure (opioid vs NSAID) and time into the models. The proportional hazards assumption was met for each outcome of interest. As an additional sensitivity analysis, we modeled the study outcomes using a logistic regression. All analyses were conducted using SAS, version 7.15 (SAS Institute Inc).

## Results

The study included 1 454 824 individuals who received publicly funded prescription opioid therapy or NSAID therapy for the first time in Ontario between March 1, 2008, and March 17, 2019 (Figure). Of these, 765 464 (52.6%) initiated therapy with a prescription opioid and 689 360 (47.4%) initiated therapy with a prescription NSAID (Table 1). Most individuals in the cohort were older adults, with 75.2% aged 65 years or older, and the cohort was evenly distributed among income quintiles. Most individuals were women (55.2%) and lived in urban settings (87.7%).

**Table 1.** Cohort Characteristics Stratified by Exposure Before Inverse Probability Treatment Weighting

Characteristic	Individuals^a^		
	Overall (N = 1 454 824)	Opioid recipients (n = 765 464)	NSAID recipients (n = 689 360)
Age group, y			
≤24	87 583 (6.0)	38 543 (5.0)	49 040 (7.1)
25-44	120 815 (8.3)	55 085 (7.2)	65 730 (9.5)
45-64	166 753 (11.5)	80 382 (10.5)	86 371 (12.5)
65-74	667 453 (45.9)	337 765 (44.1)	329 688 (47.8)
≥75	412 220 (28.3)	253 689 (33.1)	158 531 (23.0)^b^
Sex			
Female	802 749 (55.2)	406 682 (53.1)	396 067 (57.5)
Male	652 075 (44.8)	358 782 (46.9)	293 293 (42.5)
Income quintile^c^			
First	340 964 (23.4)	171 497 (22.4)	169 467 (24.6)
Second	304 594 (20.9)	158 802 (20.7)	145 792 (21.1)
Third	278 337 (19.1)	146 563 (19.1)	131 774 (19.1)
Fourth	266 248 (18.3)	142 761 (18.7)	123 487 (17.9)
Fifth	264 681 (18.2)	145 841 (19.1)	118 840 (17.2)
Urban residence	1 275 764 (87.7)	667 849 (87.2)	607 915 (88.2)
Comorbidities			
ADG score			
Median (IQR)	7 (5-9)	7 (5-10)	6 (4-9)^b^
Mean (SD)	7.03 (3.34)	7.40 (3.43)	6.61 (3.18)^b^
COPD	100 586 (6.9)	67 109 (8.5)	35 477 (5.1)^b^
Congestive heart failure	92 339 (6.4)	67 436 (8.8)	24 903 (3.6)^b^
Diabetes	348 391 (24.0)	199 178 (26.0)	149 213 (21.6)
Hypertension	818 832 (56.3)	458 449 (59.9)	360 383 (52.3)^b^
Rheumatoid arthritis	21 800 (1.5)	12 408 (1.6)	9392 (1.4)
Medication use in the past 6 mo			
Antiemetics	96 775 (6.7)	60 376 (7.9)	36 399 (5.3)
Anticonvulsants	62 099 (4.3)	35 285 (4.6)	26 814 (3.9)
Anti-Parkinson drugs	23 062 (1.6)	13 149 (1.7)	9913 (1.4)
Antihistamines	49 014 (3.4)	28 511 (3.7)	20 503 (3.0)
Antihypertensives	836 459 (57.5)	475 101 (62.1)	361 358 (52.4)^b^
Antipsychotics	72 010 (5.0)	36 388 (4.8)	35 622 (5.2)
Benzodiazepines	181 526 (12.5)	104 058 (13.6)	77 468 (11.2)
Barbiturates	2182 (0.2)	1280 (0.2)	902 (0.1)
Oral hypoglycemics	248 410 (17.1)	144 352 (18.9)	104 058 (15.1)
Muscle relaxants	177 (0.01)	97 (0.0)	80 (0.0)
Antidepressants			
SNRI	48 383 (3.3)	26 278 (3.4)	22 105 (3.2)
SSRI	137 868 (9.5)	75 746 (9.9)	62 122 (9.0)
TCA	47 008 (3.2)	26 351 (3.4)	20 657 (3.0)

Abbreviations: ADG, aggregated diagnosis group; COPD, chronic obstructive pulmonary disease; NSAID, nonsteroidal anti-inflammatory drug; SNRI, serotonin-norepinephrine reuptake inhibitor; SSRI, selective serotonin reuptake inhibitor; TCA, tricyclic antidepressant.

^a^ Data are presented as number (percentage) or individuals unless otherwise indicated.

^b^ Meaningful difference based on standardized difference greater than 0.10 compared with the opioid recipient group.

^c^ First income quintile refers to the lowest income quintile, and fifth, to the highest.

Before IPTW was applied, we found no meaningful differences in demographic characteristics between the opioid and NSAID groups with the exception of age. A higher proportion of opioid recipients were aged 75 years or older compared with NSAID recipients (33.1% vs 23.0%; standardized difference = 0.23). We also observed a higher burden of comorbidities, prescription medication use, and health care services use among opioid recipients compared with NSAID recipients before IPTW was applied (Table 1 and Table 2). The exposure groups were balanced (standardized difference for all baseline characteristics, <0.10) after application of IPTW and trimming of the cohort, and a total of 758 168 opioid recipients and 682 089 NSAID recipients were included in the weighted models (Figure).

**Table 2.** Health Care Service Use in the Year Before the Index Date Stratified by Exposure Before Inverse Probability Treatment Weighting

Health care service	Individuals		
	Overall (N = 1 454 824)	Opioid recipients (n = 765 464)	NSAID recipients (n = 689 360)
Alcohol or substance use disorder, No. (%)			
Hospitalization	3725 (0.3)	2496 (0.3)	1256 (0.2)
Emergency department visit	9079 (0.6)	4856 (0.6)	4223 (0.6)
Outpatient physician visit	19 372 (1.3)	10 182 (1.3)	9190 (1.3)
Health care provider visits, mean (SD)			
Emergency department visit for any reason	0.66 (1.54)	0.79 (1.64)	0.51 (1.39)^a^
Hospitalization for any reason	0.12 (0.45)	0.17 (0.54)	0.07 (0.31)^a^
Outpatient physician visit			
Any reason	8.83 (7.07)	9.51 (7.40)	8.09 (6.61)^a^
Mental health purposes	0.69 (2.79)	0.66 (2.75)	0.72 (2.82)
Emergency department visits for MVC	0.00 (0.05)	0.00 (0.05)	0.00 (0.06)

Abbreviations: MVC, motor vehicle collision; NSAID, nonsteroidal anti-inflammatory drug.

^a^ Meaningful difference based on standardized difference greater than 0.10 when compared with opioid recipient group.

During the 11-year study period, we recorded 28 598 person-years of follow-up for opioid recipients and 25 932 person-years of follow-up for NSAID recipients. We identified 194 ED visits for injuries relating to an MVC among drivers, of which 98 (50.5%) involved new opioid recipients (3.41 per 1000 person-years; 95% CI, 2.80-4.15 per 1000 person-years) and 96 (49.5%) involved new NSAID recipients (3.64 per 1000 person-years; 95% CI, 2.98-4.45 per 1000 person-years). We found no significant difference in the hazard of an MVC between drivers who initiated opioid therapy and those who initiated NSAID therapy (weighted hazard ratio, 0.94; 95% CI, 0.70-1.25) (Table 3).

**Table 3.** Hazard of a Motor Vehicle Collision Among Drivers in the 14 Days After New Prescription Analgesic Therapy in Ontario, Canada

Variable	Individuals, No.	Person-years of follow-up	Individuals with MVC, No. (%)	MVC rate per 1000 person-years (95% CI)	Hazard ratio (95% CI)
**Overall**					
Unadjusted estimates					
NSAID recipients	689 360	26 346.4	96.0 (49.5)	3.64 (2.98-4.45)	1 [Reference]
Opioid recipients	765 464	28 746.9	98.0 (50.5)	3.41 (2.80-4.15)	0.96 (0.72-1.27)
IPTW analysis					
NSAID recipients	678 838.1	25 932.4	92.3 (49.4)	3.56 (2.90-4.36)	1 [Reference]
Opioid recipients	758 884.2	28 598.0	94.4 (50.6)	3.30 (2.70-4.04)	0.94 (0.70-1.25)
**By dose**					
Unadjusted estimates					
NSAID recipients	689 360	26 346.4	96.0 (49.5)	3.64 (2.98-4.45)	1 [Reference]
Opioid recipients, MEQ^a^					
<50	625 751	23 546.0	77.0 (39.7)	3.27 (2.62-4.09)	0.92 (0.68-1.24)
≥50	139 694	5200.1	21.0 (10.8)	4.04 (2.64-6.19)	1.12 (0.70-1.79)
IPTW analysis					
NSAID recipients	678 838.1	25 932.4	92.3 (49.4)	3.56 (2.90-4.36)	1 [Reference]
Opioid recipients, MEQ^a^					
<50	621 587.6	23 464.9	72.4 (38.8)	3.09 (2.45-3.88)	0.88 (0.64-1.19)
≥50	137 279.3	5132.4	22.0 (11.8)	4.29 (2.83-6.50)	1.20 (0.75-1.91)

Abbreviations: IPTW, inverse probability of treatment weighting; MEQ, milligrams of morphine or equivalent; MVC, motor vehicle collision; NSAID, nonsteroidal anti-inflammatory drug.

^a^ Number of individuals in the groups that received less than 50 MEQ and 50 MEQ or greater do not add to the total number of opioid recipients as specified in the overall estimates because of variation in data availability; 19 opioid recipients were dispensed methadone for which dose information was not available in the databases.

When opioid recipients were stratified by initial daily opioid dose (ie, <50 MEQ and ≥50 MEQ), the associations between exposure and primary outcome were not significant. Among opioid recipients whose initial opioid dose was less than 50 MEQ, the weighted hazard ratio was 0.88 (95% CI, 0.64-1.19), whereas among those with an initial dose of 50 MEQ or greater, the weighted hazard ratio was 1.20 (95% CI, 0.75-1.91) (Table 3). We did not find a significant association between new opioid therapy and hazard of an MVC among passengers (eTable 3 in the Supplement). In the sensitivity analysis with study outcomes modeled using a logistic regression, the results were consistent (eTables 6 and 7 in the Supplement).

## Discussion

In this retrospective, population-based cohort study examining MVC risk after analgesia initiation, we found no significant difference in hazard of an ED visit for injuries related to an MVC between drivers starting opioid therapy and those starting NSAID therapy. Our results differ from findings in existing literature on this topic, which have reported an increase of up to 2-fold in collision risk associated with opioid use of nonspecific exposure.^19,20,21,22,23,24,25^ One potential explanation for this difference is that the literature largely consists of culpability studies (variation of case-control design)^26^ and classic case-control studies in which exposure was defined as either opioid exposed or unexposed.^19,20,21,22,23,24,25^ In those studies, the unexposed comparator group was likely to include people not experiencing pain, and therefore, findings may have been affected by the potential confounding association of pain with driving performance.^27,28^ In contrast, we used an active comparator design in which new opioid recipients were compared with individuals who received a new prescription NSAID, thereby mitigating the potential confounding association of pain with driving performance and examining the association of new analgesic therapy with hazard of collision.

Of note, the rate of injuries among drivers was substantially higher among new analgesic recipients (3.41 per 1000 person-years for opioid recipients and 3.64 per 1000 person-years for NSAID recipients) than in the general population in Ontario during a similar period (approximately 1.1 per 1000 person-years).^29^ Although we cannot exclude the possibility that differing demographic characteristics among those eligible for public drug benefits in Ontario contributed to these higher rates, these results might support other published research suggesting that pain is associated with driving ability.^27,28^ In a driving simulation study, Nilsen et al^27^ found that patients experiencing pain who were not taking opioids experienced significantly reduced reaction times and missed almost twice the number of virtual road signs compared with healthy control participants, even after controlling for age, sex, driving experience, education, and personality traits (ie, extraversion and emotional stability). Similarly, in a study from the Netherlands^28^ that compared driving performance between patients with chronic nonmalignant pain and healthy controls, the authors found that drivers with chronic pain swayed more within their lane and subjectively rated the quality of their driving significantly lower than that of healthy controls.

### Limitations

This study has limitations. First, we studied a cohort of individuals who were eligible for a public drug coverage program, which consists largely of older adults and socioeconomically disadvantaged younger individuals. Although these results are highly generalizable to the older adult population (≥65 years of age), the generalizability of our findings to groups younger than 65 years is not known. Second, we defined the cohort as residents of Ontario who were eligible for a driver’s license. It is therefore possible that some individuals who were eligible but did not acquire a driver’s license may not be at risk for the study’s outcome. However, this limitation should have affected both exposure groups equally and should not have biased our results. Furthermore, our outcome did not encompass all potential MVCs. Because of limitations in data availability, we used a visit to the ED for injuries related to an MVC as a proxy for MVCs. Therefore, we were unable to identify minor collisions that did not require medical attention and severe collisions in which fatalities were declared on the scene and thus did not involve an ED visit. This outcome definition should not have biased our results because no difference in ED visits between prescription opioid and prescription NSAID recipients was expected. In addition, our data captured instances of prescription medications dispensed, and therefore, potential exposure to over-the-counter or unregulated opioids or NSAIDs could not be identified. Although the exposure groups were balanced after application of IPTW and therefore differences before matching because of comorbidities and demographics should not affect the outcome risk, it is possible that some degree of unmeasured confounding remained given the nature of administrative data.

## Conclusions

In this large cohort study of individuals initiating analgesia therapy, we found that the hazard of an ED visit for injuries from an MVC was similar between patients starting prescription opioid therapy and those starting prescription NSAID therapy; however, this hazard was elevated compared with that in the general population. Opioids are complex medications that should be considered for use with a patient-centered approach, and the results of this study add to the wide range of considerations that may be used to inform patients, clinicians, and caregivers.

## Notes

Supplement. **eTable 1.** Diagnosis Codes for Palliative Care Services and Alcohol or Substance Use Disorder and the Corresponding Databases Each Code Can Be Found In **eTable 2.** External Cause of Injury Codes (from International Classification of Diseases 10th Revision) Used to Identify All Drivers and Passengers Who Visited the Department for Injuries Relating to a Motor Vehicle Collision (MVC) **eTable 3.** Motor Vehicle Collision Risk for Passengers in the 14 Days Following New Prescription Analgesic Therapy in Ontario, Canada; Overall and Stratified by Opioid Dose **eTable 4.** Cohort Characteristics Stratified by Exposure (Opioid Recipients vs NSAID Recipients), After IPTW Weighting **eTable 5.** Healthcare Services Utilization in the Year Prior to Index Date Stratified by Exposure (Opioid Recipients vs NSAID Recipients), After IPTW Weighting **eTable 6.** Odds of Motor Vehicle Collisions for Drivers in the 14 Days Following New Prescription Analgesic Therapy in Ontario, Canada; Overall and Stratified by Dose **eTable 7.** Odds of Motor Vehicle Collisions for Passengers in the 14 Days Following New Prescription Analgesic Therapy in Ontario, Canada; Overall and Stratified by Dose **eFigure.** Standardized Differences for Baseline Characteristics Between Opioid and NSAID Exposure Groups Before (Solid Blue Circles) and After (Empty Red Circles) Applying Inverse Probability of Treatment Weights (IPTW) to the Cohort and Trimming the Top and Bottom 0.5 Percentile of the Original Cohort

## References

[1] Centers for Disease Control and Prevention. Injuries and violence are leading causes of death. Accessed January 19, 2021. https://www.cdc.gov/injury/wisqars/animated-leading-causes.html

[2] Yao X, Skinner R, McFaull S, Thompson W. At-a-glance—2015 injury deaths in Canada. Health Promot Chronic Dis Prev Can. 2019;39(6-7):225-231. doi:10.24095/hpcdp.39.6/7.03 31210048 PMC6699611

[3] Government of Canada. Canadian motor vehicle traffic collision statistics: 2018. Accessed December 1, 2020. https://tc.canada.ca/en/road-transportation/motor-vehicle-safety/canadian-motor-vehicle-traffic-collision-statistics-2018

[4] Centers for Disease Control and Prevention. Injury prevention & control: nonfatal injury data, 2000-2018. Accessed October 6, 2020. https://www.cdc.gov/injury/wisqars/nonfatal.html

[5] Mazereeuw G, Sullivan MD, Juurlink DN. Depression in chronic pain: might opioids be responsible? Pain. 2018;159(11):2142-2145. doi:10.1097/j.pain.0000000000001305 29905651

[6] Yoshikawa A, Ramirez G, Smith ML, . Opioid use and the risk of falls, fall injuries and fractures among older adults: a systematic review and meta-analysis. *J Gerontol A Biol Sci Med Sci*. 2020;75(10):1989-1995. doi:10.1093/gerona/glaa038 32016284

[7] Centre for Addiction and Mental Health. Methadone maintenance treatment client handbook, revised. Accessed July 11, 2017. https://www.porticonetwork.ca/documents/489955/0/Methadone+Maintenance+treatment+client+handbook+PDF/e04600c3-d0c1-47e1-9a25-346a66967a77

[8] Gomes T, Redelmeier DA, Juurlink DN, Dhalla IA, Camacho X, Mamdani MM. Opioid dose and risk of road trauma in Canada: a population-based study. JAMA Intern Med. 2013;173(3):196-201. doi:10.1001/2013.jamainternmed.733 23318919

[9] Canadian Institute for Health Information. Opioid prescribing in Canada: how are practices changing? Accessed January 5, 2021. https://www.cihi.ca/sites/default/files/document/opioid-prescribing-canada-trends-en-web.pdf

[10] Drummer OH, Gerostamoulos J, Batziris H, . The involvement of drugs in drivers of motor vehicles killed in Australian road traffic crashes. Accid Anal Prev. 2004;36(2):239-248. doi:10.1016/S0001-4575(02)00153-7 14642878

[11] Findley LR, Bulloch MN. Relationship between nonsteroidal anti-inflammatory drugs and fall risk in older adults. Consult Pharm. 2015;30(6):346-351. doi:10.4140/TCP.n.2015.346 26048465

[12] McGwin G Jr, Sims RV, Pulley L, Roseman JM. Relations among chronic medical conditions, medications, and automobile crashes in the elderly: a population-based case-control study. Am J Epidemiol. 2000;152(5):424-431. doi:10.1093/aje/152.5.424 10981455

[13] Statistics Canada. Population estimates, quarterly. Accessed May 1, 2021. https://www150.statcan.gc.ca/t1/tbl1/en/tv.action?pid=1710000901

[14] ICES. Data & analytics services. Accessed September 1, 2020. https://www.ices.on.ca/DAS

[15] Mamdani M, Sykora K, Li P, . Reader’s guide to critical appraisal of cohort studies: 2. Assessing potential for confounding. BMJ. 2005;330(7497):960-962. doi:10.1136/bmj.330.7497.960 15845982 PMC556348

[16] Johns Hopkins University. The Johns Hopkins University ACG system: state of the art technology and tradition of excellence in one integrated solution. White Paper-Technical. December 2012. Accessed May 1, 2021. https://www.johnshopkinssolutions.com/wp-content/uploads/2014/04/ACG-White-Paper-Technical-Dec-2012-rev.pdf

[17] Ontario Drug Policy Research Network. ODPRN suggested calculation of opioid milligrams of morphine equivalents. Accessed August 16, 2021. https://odprn.ca/wp-content/uploads/2020/11/Opioid-Milligrams-of-Morphine-Equivalents_FINAL.pdf

[18] Busse JW, Craigie S, Juurlink DN, . Guideline for opioid therapy and chronic noncancer pain. CMAJ. 2017;189(18):E659-E666. doi:10.1503/cmaj.170363 28483845 PMC5422149

[19] Corsenac P, Lagarde E, Gadegbeku B, . Road traffic crashes and prescribed methadone and buprenorphine: a French registry-based case-control study. Drug Alcohol Depend. 2012;123(1-3):91-97. doi:10.1016/j.drugalcdep.2011.10.022 22104480

[20] Dubois S, Bédard M, Weaver B. The association between opioid analgesics and unsafe driving actions preceding fatal crashes. Accid Anal Prev. 2010;42(1):30-37. doi:10.1016/j.aap.2009.06.030 19887141

[21] Reguly P, Dubois S, Bédard M. Examining the impact of opioid analgesics on crash responsibility in truck drivers involved in fatal crashes. Forensic Sci Int. 2014;234:154-161. doi:10.1016/j.forsciint.2013.11.005 24378316

[22] Leveille SG, Buchner DM, Koepsell TD, McCloskey LW, Wolf ME, Wagner EH. Psychoactive medications and injurious motor vehicle collisions involving older drivers. Epidemiology. 1994;5(6):591-598. doi:10.1097/00001648-199411000-00006 7841240

[23] Chihuri S, Li G. Use of prescription opioids and initiation of fatal 2-vehicle crashes. JAMA Netw Open. 2019;2(2):e188081. doi:10.1001/jamanetworkopen.2018.8081 30768194 PMC6484610

[24] Monárrez-Espino J, Laflamme L, Rausch C, Elling B, Möller J. New opioid analgesic use and the risk of injurious single-vehicle crashes in drivers aged 50-80 years: a population-based matched case-control study. Age Ageing. 2016;45(5):628-634. doi:10.1093/ageing/afw115 27496939

[25] Dussault C, Brault M, Bouchard J, Lemire AM. The contribution of alcohol and other drugs among fatally injured drivers in Quebec: some preliminary results. Paper presented at Road Traffic and Psychoactive Substances. Seminar organized by the Pompidou Group, June 2003; Strasbourg, France. Accessed February 3, 2021. https://rm.coe.int/168074600f#page=215

[26] Brubacher J, Chan H, Asbridge M. Culpability analysis is still a valuable technique. Int J Epidemiol. 2014;43(1):270-272. doi:10.1093/ije/dyt142 24038716

[27] Nilsen HK, Landrø NI, Kaasa S, Jenssen GD, Fayers P, Borchgrevink PC. Driving functions in a video simulator in chronic non-malignant pain patients using and not using codeine. Eur J Pain. 2011;15(4):409-415. doi:10.1016/j.ejpain.2010.09.008 20947399

[28] Veldhuijzen DS, van Wijck AJM, Wille F, . Effect of chronic nonmalignant pain on highway driving performance. Pain. 2006;122(1-2):28-35. doi:10.1016/j.pain.2005.12.019 16495013

[29] Road Safety Research Office. Ontario road safety annual report 2019. Accessed May 1, 2021. http://www.mto.gov.on.ca/english/publications/pdfs/preliminary-2019-orsar-selected-statistics.pdf

