# Supplementary material for: New Opioid Use and Risk of Emergency Department Visits Related to Motor Vehicle Collisions in Ontario, Canada
Source: JAMA Netw Open. 2021 Nov 11;4(11):e2134248. doi: 10.1001/jamanetworkopen.2021.34248 (PMC8586904; doi:10.1001/jamanetworkopen.2021.34248)
Supplement: Supplement. — eTable 1. Diagnosis Codes for Palliative Care Services and Alcohol or Substance Use Disorder and the Corresponding Databases Each Code Can Be Found In eTable 2. External Cause of Injury Codes (from International Classification of Diseases 10th Revision) Used to Identify All Drivers and Passengers Who Visited the Department for Injuries Relating to a Motor Vehicle Collision (MVC) eTable 3. Motor Vehicle Collision Risk for Passengers in the 14 Days Following New Prescription Analgesic Therapy in Ontario, Canada; Overall and Stratified by Opioid Dose eTable 4. Cohort Characteristics Stratified by Exposure (Opioid Recipients vs NSAID Recipients), After IPTW Weighting eTable 5. Healthcare Services Utilization in the Year Prior to Index Date Stratified by Exposure (Opioid Recipients vs NSAID Recipients), After IPTW Weighting eTable 6. Odds of Motor Vehicle Collisions for Drivers in the 14 Days Following New Prescription Analgesic Therapy in Ontario, Canada; Overall and Stratified by Dose eTable 7. Odds of Motor Vehicle Collisions for Passengers in the 14 Days Following New Prescription Analgesic Therapy in Ontario, Canada; Overall and Stratified by Dose eFigure. Standardized Differences for Baseline Characteristics Between Opioid and NSAID Exposure Groups Before (Solid Blue Circles) and After (Empty Red Circles) Applying Inverse Probability of Treatment Weights (IPTW) to the Cohort and Trimming the Top and Bottom 0.5 Percentile of the Original Cohort [file jamanetwopen-e2134248-s001.pdf]

Supplemental Online Content

Guan Q, McCormack D, Juurlink DN, Bronskill SE, Wunsch H, Gomes T. New opioid use and risk of emergency department visits related to motor vehicle collisions in Ontario, Canada. *JAMA Netw Open.* 2021;4(11):e2134248. doi:10.1001/jamanetworkopen.2021.34248

eTable 1. Diagnosis Codes for Palliative Care Services and Alcohol or Substance Use Disorder and the Corresponding Databases Each Code Can Be Found In

eTable 2. External Cause of Injury Codes (from International Classification of Diseases 10th Revision) Used to Identify All Drivers and Passengers Who Visited the Department for Injuries Relating to a Motor Vehicle Collision (MVC)

eTable 3. Motor Vehicle Collision Risk for Passengers in the 14 Days Following New Prescription Analgesic Therapy in Ontario, Canada; Overall and Stratified by Opioid Dose

eTable 4. Cohort Characteristics Stratified by Exposure (Opioid Recipients vs NSAID Recipients), After IPTW Weighting

eTable 5. Healthcare Services Utilization in the Year Prior to Index Date Stratified by Exposure (Opioid Recipients vs NSAID Recipients), After IPTW Weighting

eTable 6. Odds of Motor Vehicle Collisions for Drivers in the 14 Days Following New Prescription Analgesic Therapy in Ontario, Canada; Overall and Stratified by Dose

eTable 7. Odds of Motor Vehicle Collisions for Passengers in the 14 Days Following New Prescription Analgesic Therapy in Ontario, Canada; Overall and Stratified by Dose

eFigure. Standardized Differences for Baseline Characteristics Between Opioid and NSAID Exposure Groups Before (Solid Blue Circles) and After (Empty Red Circles) Applying Inverse Probability of Treatment Weights (IPTW) to the Cohort and Trimming the Top and Bottom 0.5 Percentile of the Original Cohort

This supplemental material has been provided by the authors to give readers additional information about their work.

© 2021 Guan Q et al. *JAMA Network Open.*

e-Table 1. Diagnosis codes for palliative care services and alcohol or substance use disorder and the corresponding databases each code can be found in.

Database	Codes
Palliative Care Codes	
Ontario Health Insurance Plan Database (OHIP)	A945, B998, C945, C882, C982, K023, W872, W882, W972, W982
CIHI Discharge Abstract Database	PATSERV=58
Alcohol Use Disorder or Substance Use Disorder	
OHIP	3030, 304
CIHI-DAD and CIHI National Ambulatory Care Reporting System	F10-F17

e-Table 2. External cause of injury codes (from International Classification of Diseases 10th Revision) used to identify all drivers and passengers who visited the emergency department for injuries relating to a motor vehicle collision (MVC). An individual is classified as having an emergency department visit for an MVC if these codes are identified in any of their emergency department diagnoses.

Vehicle Involved	Driver	Passenger
Motorcycle	V200, V202, V204, V209, V210, V212, V214, V219, V220, V222, V224, V229, V230, V232, V234, V239, V240, V242, V244, V249, V250, V252, V254, V259, V260, V262, V264, V269, V270, V272, V274, V279, V280, V282, V284, V289, V290, V292, V293, V294, V296, V298, V299	V201, V205, V211, V215, V221, V225, V231, V235, V241, V245, V251, V255, V261, V265, V271, V275, V281, V285, V291, V295
Three-wheeled motor vehicle	V300, V305, V310, V315, V320, V325, V330, V335, V340, V345, V350, V355, V360, V365, V370, V375, V380, V385, V390, V394	V301, V306, V311, V316, V321, V326, V331, V336, V341, V346, V351, V356, V361, V366, V371, V376, V381, V386, V391, V395
Car	V400, V405, V410, V415, V420, V425, V430, V435, V440, V445, V450, V455, V460, V465, V470, V475, V480, V485, V490, V494	V401, V406, V411, V416, V421, V426, V431, V436, V441, V446, V451, V456, V461, V466, V471, V476, V481, V486, V491, V495
Van or pick-up truck	V500, V505, V510, V515, V520, V525, V530, V535, V540, V545, V550, V555, V560, V565, V570, V575, V580, V585, V590, V594	V501, V506, V511, V516, V521, V526, V531, V536, V541, V546, V551, V556, V561, V566, V571, V576, V581, V586, V591, V595
Heavy transport vehicle	V600, V605, V610, V615, V620, V625, V630, V635, V640, V645, V650, V655, V660, V665, V670, V675, V680, V685, V690, V694	V601, V606, V611, V616, V621, V626, V631, V636, V641, V646, V651, V656, V661, V666, V671, V676, V681, V686, V691, V695

© 2021 Guan Q et al. *JAMA Network Open.*

Bus	V700, V705, V710, V715, V720, V725, V730, V735, V740, V745, V750, V755, V760, V765, V770, V775, V780, V785, V790, V794	V701, V706, V711, V716, V721, V726, V731, V736, V741, V746, V751, V756, V761, V766, V771, V776, V781, V786, V791, V795
Industrial, agricultural, construction or all-terrain vehicle	V830, V835, V840, V845, V850, V855, V860, V865	V831, V836, V841, V846, V851, V856, V861

© 2021 Guan Q et al. *JAMA Network Open.*

e-Table 3. Motor vehicle collision risk for passengers in the 14 days following new prescription analgesic therapy in Ontario, Canada; overall and stratified by opioid dose

	**Total Number of Individuals**	**Total Number of Person-Years of Follow-Up**	**Number of Individuals with MVC**	**MVC Rate per 1,000 Person-Years (95% CI)**	**Hazard Ratio (95% CI)**
Overall					
**Unadjusted Estimates**					
NSAID Recipients	689,360	26,347.3	47	1.78 (1.34 - 2.37)	1.00 [Reference]
Opioid Recipients	765,464	28,747.5	55	1.91 (1.47 - 2.49)	1.09 (0.74 - 1.62)
**IPTW Analysis**					
NSAID Recipients	678,837.6	25,933.3	47.0	1.81 (1.36 - 2.41)	1.00 [Reference]
Opioid Recipients	758,884.3	28,598.6	53.4	1.87 (1.43 - 2.44)	1.03 (0.70 - 1.52)
By Dose					
**Unadjusted Estimates**					
NSAID Recipients	689,360	26,347.3	47	1.78 (1.34 - 2.37)	1.00 [Reference]
Opioid Recipients					
<50MEQ *	625,751	23,294.7	42	1.80 (1.33 - 2.44)	1.03 (0.68 – 1.56)
≥50MEQ *	139,694	5,140.8	13	2.53 (1.47 - 4.35)	1.41 (0.76 – 2.61)
**IPTW Analysis**					
NSAID Recipients	678,837.6	25,933.3	47.0	1.81 (1.36 - 2.41)	1.00 [Reference]
Opioid Recipients					
<50MEQ *	621,588.5	23,465.5	40.6	1.73 (1.27 - 2.35)	0.97 (0.63 – 1.47)
≥50MEQ *	137,279.4	5,131.6	12.8	2.49 (1.44 - 4.31)	1.37 (0.74 – 2.54)

** Number of individuals in <50MEQ group and ≥50MEQ group do not add up to total number of opioid recipients as specified in the "Overall" estimates due to data availability. 19 opioid recipients were dispensed methadone for which dose information is not available in our databases.*

Abbreviations: CI, confidence interval; IPTW, inverse probability of treatment weighting; MEQ, milligrams of morphine or equivalent; MVC, motor vehicle collision; NSAID, non-steroidal anti-inflammatory drug

© 2021 Guan Q et al. *JAMA Network Open.*

e-Table 4. Cohort characteristics stratified by exposure (opioid recipients vs. NSAID recipients), after IPTW weighting

	Opioid Recipients	NSAID Recipients
	N=758,884	N=678,838
**Demographics**		
Age Categories, No. (%)		
≤24 Years	46,886.9 (6.2)	42,660.2 (6.3)
25-44 Years	64,353.6 (8.5)	58,420 (8.6)
45-64 Years	87,286.1 (11.5)	78,571.5 (11.6)
65-74 Years	348,838 (46.0)	313,193 (46.1)
≥75 Years	211,520 (27.9)	185,993 (27.4)
Females, No. (%)	419,875 (55.3)	376, 536 (55.5)
Income Quintile, No. (%)		
1	178,419 (23.5)	160,247 (23.6)
2	158,852 (20.9)	142,155 (20.9)
3	145,026 (19.1)	129,619 (19.1)
4	138,652 (18.3)	123,750 (18.2)
5	137,935 (18.2)	123,067 (18.1)
Urban Residence	665,360 (81.8)	555,771 (8.18)
**Comorbidities**		
ADG Score, mean	7.00	6.92
COPD, No. (%)	53,379.2 (7.0)	40,688.4 (6.0)
Congestive Heart Failure, No. (%)	52,643.4 (6.9)	29,128 (4.3)[1]
Diabetes, No. (%)	179,324 (23.6)	158,947 (23.4)
Hypertension, No. (%)	423,327 (55.8)	377,562 (55.6)
Rheumatoid Arthritis, No. (%)	11,448.3 (1.5)	9,662.6 (1.4)
**Medication Use in the Past 6 Months, No (%)**		
Antiemetics	48,973.9 (6.5)	42,808.4 (6.3)
Anticonvulsants	31,895.8 (4.2)	28,5547.2 (4.2)
Anti-Parkinson's drugs	11,859.8 (1.6)	10,609.3 (1.6)
Antihistamines	25,200.8 (3.3)	22,348.7 (3.3)
Antihypertensives	432,585 (57.0)	384,461 (56.6)
Antipsychotics	37,886.9 (5.0)	34,434.7 (5.1)
Benzodiazepines	93,687.8 (12.4)	83,563.3 (12.3)
Barbiturates	1,111.9 (0.2)	998.0 (0.2)
Oral hypoglycemics	127,716 (16.8)	112,974 (16.6)
Muscle relaxants	94.4 (0.0)	85.4 (0.0)
SNRI antidepressants	25,233.1 (3.3)	22,674.5 (3.3)
SSRI antidepressants	71,492.8 (9.4)	64,003.5 (9.4)
Tricyclic antidepressants	24,335.8 (3.2)	21,738.4 (3.2)

[1] Meaningful difference based on standardized difference >0.10 when compared to Opioid Recipient group

Abbreviations: ADG, Aggregated Diagnosis Group; COPD, chronic obstructive pulmonary disease; IQR, interquartile range; NSAID, non-steroidal anti-inflammatory drug; SNRI, serotonin-norepinephrine reuptake inhibitor; SSRI, selective serotonin reuptake inhibitor

© 2021 Guan Q et al. *JAMA Network Open.*

e-Table 5. Healthcare services utilization in the year prior to index date stratified by exposure (opioid recipients vs. NSAID recipients), after IPTW weighting

	**Opioid Recipients**	**NSAID Recipients**
	**N=758,884**	**N=678,838**
Alcohol/Substance Use Disorder, No. (%)		
Hospitalization	1,749.3 (0.2)	1,582.2 (0.2)
Emergency Department Visit	4,712.4 (0.6)	4,426.2 (0.7)
Outpatient Physician Visit	10,135.9 (1.3)	9,341 (1.4)
Mean Number of Healthcare Provider Visits		
Emergency Department Visits (any reason)	0.6	0.6
Hospitalizations (any reason)	0.1	0.1
Outpatient Physician Visits (any reason)	8.7	8.7
Outpatient Physician Visits for Mental Health Purposes	0.7	0.7
Emergency Department Visits for MVC	0.00	0.00

[1]*Meaningful difference based on standardized difference >0.10 when compared to Opioid Recipient group*
Abbreviations: MVC, motor vehicle collision; NSAID, non-steroidal anti-inflammatory drug; SD, standard deviation

© 2021 Guan Q et al. *JAMA Network Open.*

**e-Table 6**. Odds of motor vehicle collisions for drivers in the 14 days following new prescription analgesic therapy in Ontario, Canada; overall and stratified by dose		
	**Number of Events**	**Odds Ratios**
Overall		
**Unadjusted**		
NSAID Recipients	96	1.00 [reference]
Opioid Recipients	98	0.94 (0.71 – 1.25)
**IPTW Analysis**		
NSAID Recipients	92.3	1.00 [reference]
Opioid Recipients	94.4	0.92 (0.69 – 1.23)
By Dose		
**Unadjusted**		
NSAID Recipients	96	1.00 [reference]
<50 MEQ	77	0.91 (0.67 – 1.22)
≥50 MEQ	21	1.09 (0.68 – 1.75)
**IPTW Analysis**		
NSAID Recipients	92.3	1.00 [reference]
<50 MEQ	72.4	0.87 (0.64 – 1.18)
≥50 MEQ	22.0	1.18 (0.74 – 1.87)

**e-Table 7**. Odds of motor vehicle collisions for passengers in the 14 days following new prescription analgesic therapy in Ontario, Canada; overall and stratified by dose		
	**Number of Events**	**Odds Ratios**
Overall		
**Unadjusted**		
NSAID Recipients	47	1.00 [reference]
Opioid Recipients	55	1.08 (0.31 – 1.59)
**IPTW Analysis**		
NSAID Recipients	47	1.00 [reference]
Opioid Recipients	53.4	1.03 (0.69 – 1.52)
By Dose		
**Unadjusted**		
NSAID Recipients	47	1.00 [reference]
<50 MEQ	42	1.01 (0.67 – 1.53)
≥50 MEQ	13	1.38 (0.75 – 2.55)
**IPTW Analysis**		
NSAID Recipients	47	1.00 [reference]
<50 MEQ	40.6	0.96 (0.63 – 1.45)
≥50 MEQ	12.8	1.34 (0.72 – 2.48)

© 2021 Guan Q et al. *JAMA Network Open.*

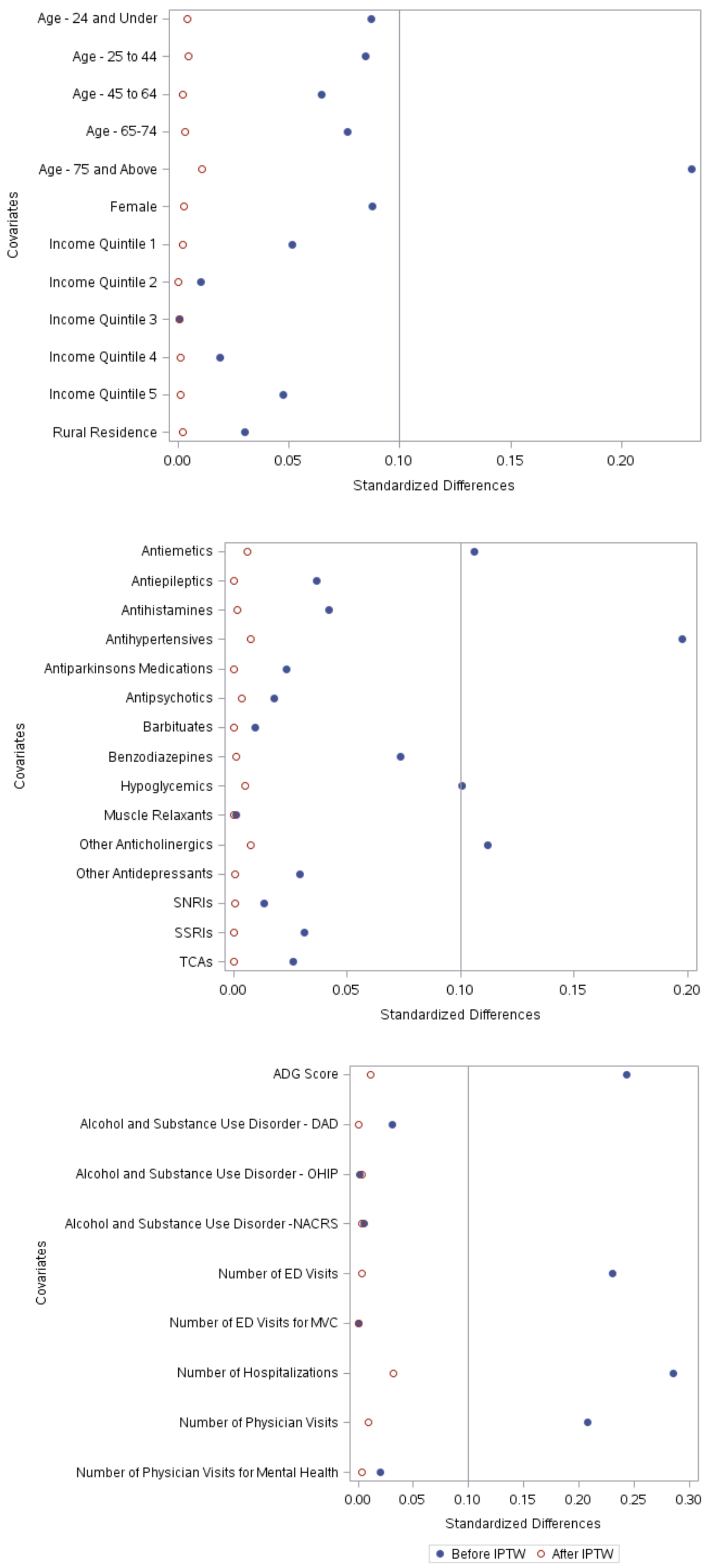

e-Figure 1. Standardized differences for baseline characteristics between opioid and NSAID exposure groups before (solid blue circles) and after (empty red circles) applying inverse probability of treatment weights (IPTW) to the cohort and trimming the top and bottom 0.5 percentile of the original cohort.

© 2021 Guan Q et al. *JAMA Network Open.*
